# Green Sol–Gel Synthesis of Iron Oxide Nanoparticles for Magnetic Hyperthermia Applications

**DOI:** 10.3390/pharmaceutics16121578

**Published:** 2024-12-11

**Authors:** Juliana Jesus, Joana Regadas, Bárbara Costa, João Carvalho, Ana Pádua, Célia Henriques, Paula I. P. Soares, Sílvia Gavinho, Manuel A. Valente, Manuel P. F. Graça, Sílvia Soreto Teixeira

**Affiliations:** 1 i3N and Department of Physics, University of Aveiro, Campus Universitário de Santiago, 3810-193 Aveiro, Portugal; julianajesus@ua.pt (J.J.); joanaregadas@ua.pt (J.R.); barbaracostaa@ua.pt (B.C.); jpfc@ua.pt (J.C.); silviagavinho@ua.pt (S.G.); mav@ua.pt (M.A.V.); mpfg@ua.pt (M.P.F.G.); 2 i3N/CENIMAT, Physics Department, NOVA School of Science and Technology, Campus de Caparica, NOVA University Lisbon, 2829-516 Caparica, Portugal; as.padua@fct.unl.pt (A.P.); crh@fct.unl.pt (C.H.); 3 i3N/CENIMAT, Departamento de Ciência dos Materiais, NOVA School of Science and Technology, Campus de Caparica, NOVA University Lisbon, 2829-516 Caparica, Portugal; pi.soares@fct.unl.pt

**Keywords:** magnetite, nanoparticles, sol–gel, coconut water powder, cancer, magnetic hyperthermia

## Abstract

Background/Objectives: The unique properties of iron oxide nanoparticles have attracted significant interest within the biomedical community, particularly for magnetic hyperthermia applications. Various synthesis methods have been developed to optimize these nanoparticles. Methods: In this study, we employed a powdered coconut water (PCW)-assisted sol–gel method to produce magnetite nanoparticles for the first time. A comprehensive analysis of the thermal (differential thermal analysis and thermogravimetry), structural (X-ray diffraction), morphological (scanning electron microscopy with energy dispersive spectroscopy), magnetic (vibrating sample magnetometer and hyperthermia), and biological (cytotoxicity essays) properties was conducted to assess their potential for magnetic hyperthermia. Results: Samples heat-treated at 700 °C and 400 °C (washed powder) for 4 h under argon presented only magnetite in their composition. The micrometer-sized particles exhibited ferrimagnetic behavior, with saturation magnetization values of 37, 76, and 10 emu/g and specific absorption rates (SAR) of 27.1, 19.9, and 14.1 W/g, respectively, for treatments at 350 °C (48 h), 700 °C (4 h), and 400 °C (washed powder, 4 h) under an argon atmosphere. Biological tests showed no cytotoxicity below 10 mg/mL. Conclusions: The findings highlight the potential of PCW-assisted synthesis as a sustainable and efficient strategy for producing pure magnetite, with powder washing preceding the heat treatment enabling the attainment of this phase at lower temperatures. Nevertheless, the micrometer-scale dimensions is observed in the morphological analysis limit their suitability for biomedical applications.

## 1. Introduction

Magnetic hyperthermia is an emerging cancer treatment that shows great potential, particularly when combined with conventional therapies like chemotherapy or radiation therapy [1]. In this treatment, magnetic nanoparticles are exposed to an external alternating magnetic field, converting electromagnetic energy into heat through energy dissipation mechanisms such as Néel and Brownian relaxation. This raises the temperature inside the tumor, typically to 40–46 °C, killing cancer cells or making them more vulnerable to conventional therapies [2]. Due to their higher metabolic rates, cancer cells are more susceptible to heat than healthy cells, which allows for minimizing damage to surrounding normal tissues [3].

Iron oxide nanoparticles, particularly magnetite (Fe_3_O_4_) and maghemite (γ-Fe_2_O_3_), are widely employed in magnetic hyperthermia due to their advantageous characteristics. These nanoparticles offer excellent chemical stability, ease of surface functionalization, non-toxicity, high biocompatibility [1], superparamagnetic behavior at sizes below 20 nm [4], and easy assimilation within the body [4,5]. The last point is related to the iron ions that result from the dissolution process, which can be stored in ferritin [4,5].

In magnetic hyperthermia, the capacity of magnetic nanoparticles to generate and release heat is crucial for effective treatment. The performance of these nanoparticles can be evaluated using the specific absorption rate (SAR), defined as the heat loss per unit mass of magnetic material. SAR depends on the material’s intrinsic characteristics, including magnetic properties, size, morphology, and aggregation status [1].

Selecting the appropriate synthesis method is important to produce magnetic nanoparticles with the desired size, shape, structure, colloidal stability, and magnetic characteristics [6]. The most used techniques to synthesize iron oxide nanoparticles include coprecipitation [7,8,9], sol–gel [10,11,12], thermal decomposition [13], hydrothermal and solvothermal synthesis [14,15], auto-combustion [16,17], and microemulsion [18,19]. However, these methods typically require high temperatures, expensive equipment, and environmentally hazardous solvents. Recognizing the need for more sustainable and cost-effective approaches, the scientific community is increasingly focused on developing greener synthesis methods. In this context, a sol–gel method mediated by powdered coconut water was selected for synthesizing magnetite nanoparticles. Coconut water, derived from a natural and sustainable source, is composed of a mixture of water and various organic and inorganic compounds [20]. Its main constituents are sugars, particularly sucrose and glucose [21]. Coconut water also has free amino acids and amino acids integrated into proteins. Among them, alanine, arginine, cysteine, and serine are the most prevalent [20]. The use of coconut water as a solvent for the dissolution of the metal precursors allows better control of particle size compared to water, as demonstrated by Manali et al. [22]. However, its complex and variable chemical composition makes the exact process of stabilization and particle formation difficult to understand [21]. Some authors believe that amino acids and proteins are capable of binding/complexing to metal cations from the precursors and forming stable micelles [20,21,22]. Amino acids contain functional groups that can act as ligands and form coordinate bonds with metal ions [21]. Moreover, the large protein chains present in coconut water result in steric hindrance when the ions are complexed [22]. This promotes a uniform distribution within the colloidal solution and control over particle size and shape [20,21,22]. Additionally, sugars also present an important role as chelating agents and as fuel during the calcination process [21]. Thus, the key benefits of employing coconut water include (1) respecting green chemistry principles, (2) its role as a natural polymerization agent, (3) the presence of high sugar concentration, (4) its abundant availability in nature, (5) its renewability, (6) cost-effectiveness, (7) industrial-scale availability, and (8) a straightforward production process for the powdered form [20,23]. The use of powdered coconut water instead of fresh or bottled coconut water combines these benefits with the possibility of precisely controlling the concentration of the solution of powdered coconut water. This capability ensures the method’s reproducibility [20].

This eco-friendly method has been successfully used to produce magnetic nanoparticles, such as doped yttrium oxide [22,24,25], nickel ferrite (NiFe_2_O_4_) [23,26,27], niobium oxide (Nb_2_O_5_) [28], and barium titanate (BaTiO_3_) [21]. However, no article using this technique to synthesize magnetite nanoparticles has been found in the literature.

The main objective of this study is to assess the viability of synthesizing magnetite particles using an adapted sol–gel method. In this process, magnetite powders were prepared using iron chlorides as raw materials and powdered coconut water as a surfactant. At the end of this process, the samples were subjected to calcination in an air atmosphere or a non-oxidizing atmosphere. The non-oxidizing atmosphere was employed to prevent the oxidation of magnetite [29]. Finally, to ascertain the suitability of the produced samples for magnetic hyperthermia, they were subjected to characterization methods, including differential thermal analysis (DTA) and thermogravimetry (TG), X-ray diffraction (XRD), scanning electron microscopy (SEM), energy dispersive spectroscopy (EDS), vibrating sample magnetometer (VSM), magnetic hyperthermia assays, and cytotoxicity analysis.

## 2. Materials and Methods

### 2.1. Preparation of Fe_3_O_4_ Powders

Magnetite powders were prepared through a sol–gel proteic route, previously described by Teixeira S. et al. [20]. For this process, iron (III) chloride hexahydrate (FeCl_3_·6H_2_O) and iron (II) chloride (FeCl_2_) (Sigma-Aldrich, Munich, Germany) were employed as reagents, and PCW was used as solvent.

First, an aqueous solution of powdered coconut water (*Cocos nucifera L.*) was prepared with a concentration of 0.59 mol/dm^3^. This value corresponds to the critical micelle concentration as determined by Costa B. et al. [30]. The reagents were weighted considering a stoichiometric ratio of 2 mol Fe(III):1 mol Fe(II) and individually dissolved in coconut water. Then, they were combined, resulting in an exothermic reaction. Subsequently, the sample was placed on a heating plate under magnetic stirring for 2 h at 80 °C. The temperature was then increased to 100 °C for about 3 h to reach a viscous gel. Throughout the procedure, pH values were recorded, and a change in the trend was noted. This served as a control method to validate the occurrence of a chemical transformation during the process, where Sol becomes a 3D three-dimensional network, Gel. To remove all the solvent, the sample was subjected to a heat treatment at 250 °C for 1 h, and the resulting sample was then manually ground to obtain a fine dark powder.

Two sample groups were prepared: the first remained unwashed (base powder) and the other was washed with deionized water (washed powders). The washing process aimed to remove chlorides and nutrients from the coconut water precursor, such as potassium and calcium, since these are soluble in water. Finally, both sample groups were submitted to various heat treatments at temperatures ranging 250–1000 °C, with different time intervals (4, 24, and 48 h), using a heating rate of 5 °C/min under air or an inert argon atmosphere.

**Figure 1:**
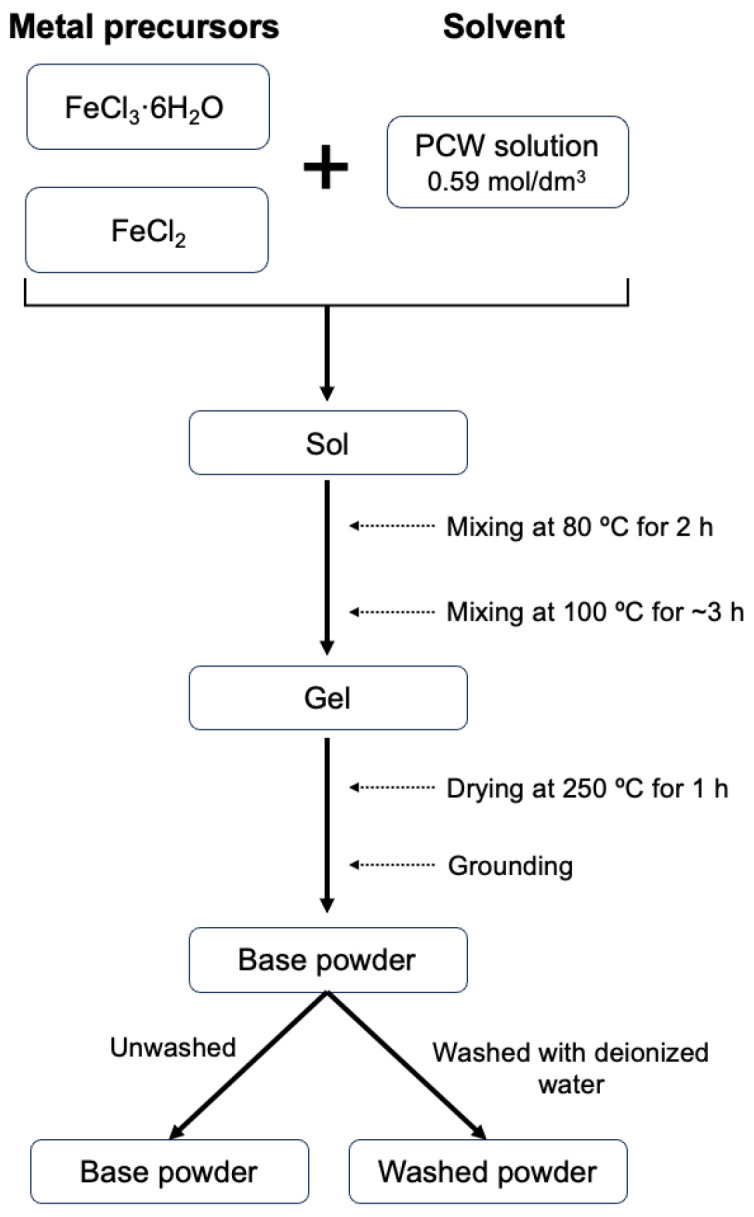
Scheme of experimental procedure for PCW-assisted sol–gel method.

The scheme represented in Figure 1 shows the flow chart for the biogenic sol–gel experimental procedure method:

### 2.2. Thermal Characterization

To investigate mass variations and thermal phenomena, facilitating the identification of suitable heat treatment temperatures, DTA/TG measurements were performed. The Hitachi (Tokyo, Japan) STA7300 was used in a temperature range from room temperature up to 1300 °C, with a heating rate of 5 °C/min, and under an argon atmosphere.

### 2.3. Structural Characterization

Structural characterization and phase identification were accomplished by X-ray diffraction (XRD). The diffractograms were obtained using an AERIS diffractometer from Malvern Panalytical (Malvern, UK), which uses CuKα radiation (λ = 1.54 Å). The X’Pert HighScore PANalytical program allowed the Rietveld refinement.

### 2.4. Morphological Characterization

Morphology analysis was conducted through SEM utilizing a TESCAN (Brno, Czechia) Vega 3 microscope. Before the observation, all the samples were coated with carbon to enhance the surface conductivity. The ImageJ (Bethesda, MD, USA) 1.54g software was used to obtain the average grain size from the SEM images. Additionally, elemental analysis was carried out using a Bruker (Billerica, MA, USA) EDS (energy dispersive spectroscopy) system coupled to the microscope. All measurements were taken at a voltage of 25 kV.

### 2.5. Magnetic Characterization

The magnetic properties of each sample were acquired using a VSM from Cryogenic. This method allowed the determination of magnetic susceptibility (for temperatures of 5 up to 300 K under a magnetic field of 100 mT) and hysteresis loop (at temperatures of 5 and 300 K using a magnetic field up to 10 T).

Magnetic hyperthermia measurements were carried out to evaluate the heating capacity of each sample when exposed to a magnetic external field. For this, a concentration of 10 mg/mL of each sample was placed under the influence of a magnetic field with an amplitude of 300 G (~24 kA/m) and a frequency of 338.4 kHz for 10 min. These measurements were performed using the nB nanoScale Biomagnetics D5 Series (Zaragoza, Spain). Before the measurement, each sample was dispersed in 1 mL of ultrapure water and subjected to an ultrasonic bath.

### 2.6. Cytotoxicity Analysis

Cytotoxicity assessment followed the methodology employed in the study by Regadas et al. [31]. Cell viability evaluation was conducted according to the “ISO 10993-5 Biological evaluation of medical devices—Part 5: Test for in vitro cytotoxicity” standard [32] using the human osteosarcoma cell line Saos-2 (from American Type Culture Collection (ATCC HTB-85)) and the extract method. The cells were seeded in 96-well tissue culture microplates, with approximately 9 kcells/well placed in 100 μL of the medium, at 37 °C in an atmosphere containing 5% CO_2_ for 24 h. All samples were previously sterilized at 120 °C for 2 h and then incubated in McCoy medium to obtain the extract with an initial concentration of 20 mg/mL. The resulting extracts were incubated for 24 h at 37 °C in an atmosphere containing 5% CO_2_. After 24 h, the medium that was in contact with the powder was removed and filtered with a 0.22 μm filter. Then, the culture medium from the seeded cells was removed and substituted with filtered extracts with sequential dilutions of 10, 5, and 2.5 mg/mL. For each concentration, 6 replicates were prepared. After 48 h of contact between the extract and the cells, a solution consisting of a 1:1 mixture of resazurin and culture medium was added and incubated for 3 h. Living cells metabolize resazurin, producing resorufin, which has a pink color, while in the absence of cells, resazurin maintains a blue color. The absorbance of each well was measured at wavelengths of 570 nm and 600 nm. This experiment needed two cell controls, a positive and a negative control. In the positive control, cells were treated with 10% DMSO to promote cell death. The negative control was based on untreated cells. Cell viability is given by the following expression:

(1) $$Cellviability\left(\%\right)=\frac{{OD}_{570-600}Sample}{{OD}_{570-600}Negative\;control}\times 100$$

## 3. Results and Discussion

### 3.1. Thermal Analysis

**Figure 2:**
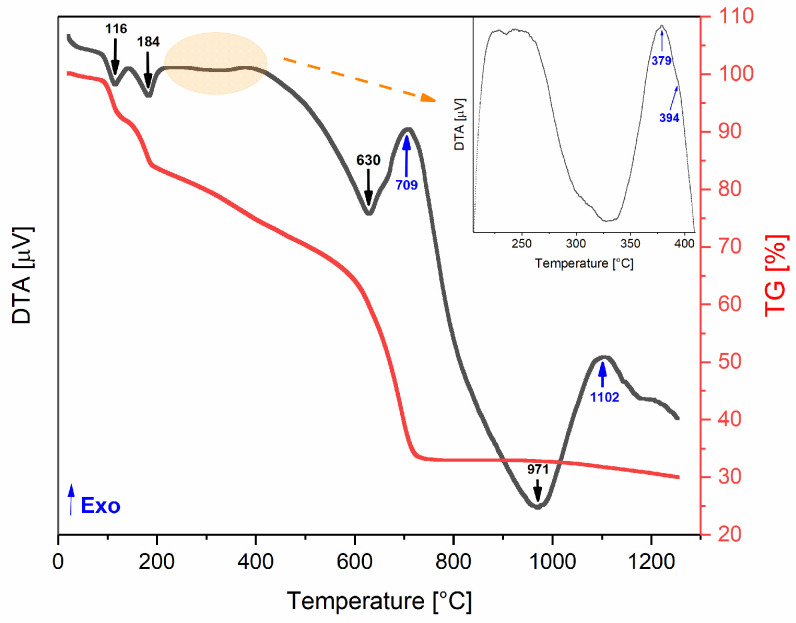
DTA/TG thermograms of the base powder.

The differential thermal (DTA) and thermogravimetric (TG) analyses of the base powder, conducted from room temperature to 1300 °C, are shown in Figure 2. The TG curve demonstrates a mass loss of approximately 70%, which occurs in four main stages. The first loss occurs between 90 and 145 °C (endothermic process) and may be associated with weakly bound water molecules (hydration). The second event occurs within the temperature range of 145 to 190 °C (endothermic process) and can be attributed to the mass loss from strongly bound water molecules (dehydroxylation). The last two mass losses occur between 190 and 720 °C, which can be attributed to the decomposition of organic chains from the precursor powder of coconut water [18] and the decomposition of chlorides from reagents (FeCl_3_·6H_2_O and FeCl_2_) [33].

An exothermic event without corresponding weight loss may indicate structural changes, such as the formation of a new crystalline phase [20]. This makes the exothermic peaks observed around 379, 394, and 709 °C particularly interesting, as they could be linked to the formation of the desired magnetite phase. At approximately 700 °C, the total mass loss is achieved (≈70%), so this temperature was established as the limit for the heat treatments. Accordingly, the selected heat treatment temperatures were 300, 350, 400, and 700 °C.

**Figure 3:**
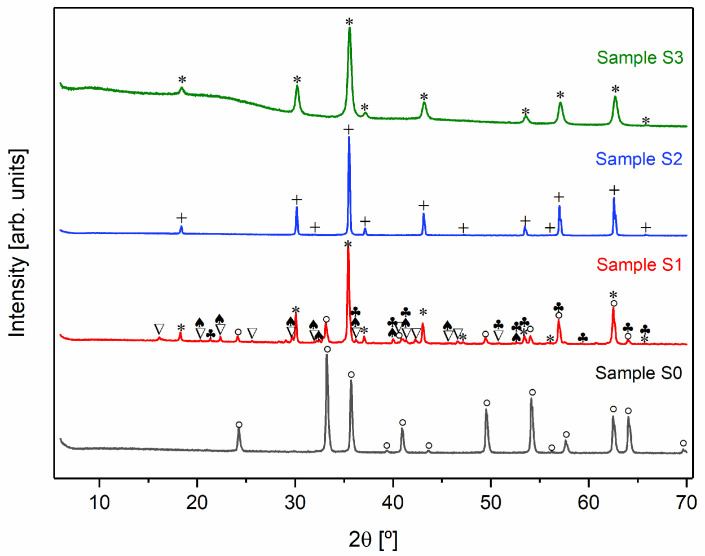
X-ray diffractograms of all samples (+ Fe_3_O_4_ monoclinic; * Fe_3_O_4_ cubic; ∇ FeCl_2_·4H_2_O monoclinic; ° α-Fe_2_O_3_; ♣ FeO(OH); ♠ FeCl_2_· 4H_2_O unknown).

Taking into account the structural results (Figure 3), namely, the detection of the Fe_3_O_4_ crystal phase, the heat treatment conditions of the most relevant samples are described in Table 1.

### 3.2. Structural Characterization

Figure 3 shows the X-ray diffractograms of the heat-treated samples and Table 2 presents the phase’s composition and their respective weight percentage in each sample.

To synthesize Fe_3_O_4_, it is necessary to carry out the heat treatment in an inert argon atmosphere to prevent the oxidation of Fe^2^⁺ to Fe^3^⁺. In the presence of oxygen, Fe_3_O_4_ tends to transform into Fe_2_O_3_ [29,34,35]. This is evident when comparing samples S0 and S2. The X-ray diffraction pattern of sample S0, which was treated in an oxygen-rich environment, indicates the presence of α-Fe_2_O_3_. In contrast, sample S2, which underwent the same heat treatment but in an argon atmosphere, displays the formation of the desired crystalline Fe_3_O_4_ phase.

As illustrated in Figure 3, all samples treated in an inert atmosphere (S1, S2, and S3) displayed diffraction peaks indicative of magnetite (Fe_3_O_4_) [36]. In addition to cubic Fe_3_O_4_, the S1 diffractogram revealed the presence of other crystalline phases. In contrast, samples S2 and S3 demonstrated the formation of pure Fe_3_O_4_, which can be associated with the exothermic peaks observed at 709 and 379 °C, respectively, in the DTA/TG thermograms (Figure 2). However, the crystallographic structure of the magnetite differed between the samples. Sample S2 exhibited a monoclinic Fe_3_O_4_ phase, while sample S3 formed a cubic Fe_3_O_4_ structure.

An analysis of the diffractograms of samples S1 and S2, in conjunction with the data presented in Table 2, indicates that chloride compounds undergo sublimation as temperature increases.

Lastly, the washing process employed on sample S3 resulted in the absence of iron chlorides in the XRD diffractograms. This outcome indicates that this alternative approach removes chlorides, enabling the formation of pure magnetite at a lower temperature. Nevertheless, despite the absence of chlorides in XRD, there is still a possibility that they may exist in the sample in an amorphous form. Furthermore, sample S3 displayed reduced crystallinity, as evidenced by broader diffraction peaks. The observed reduction in crystallinity may be attributed to the washing process, which not only eliminates chlorides but also removes some iron content.

**Figure 4:**
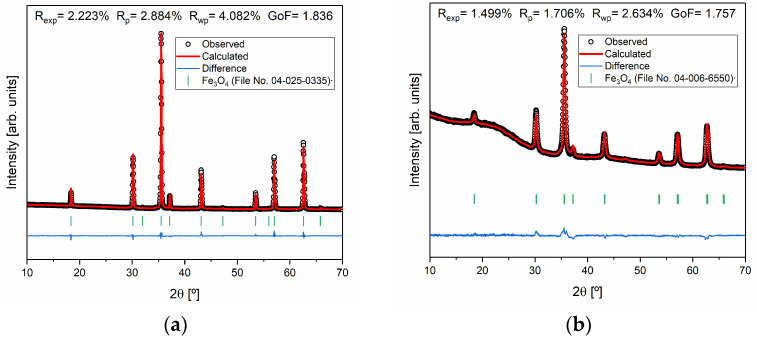
Rietveld refinement of the samples (**a**) S2 and (**b**) S3 and agreement indexes.

To better investigate the samples that presented only magnetite (S2 and S3) in their constitution, Rietveld refinement was performed, as shown in Figure 4. Considering the parameters obtained, it is possible to conclude that the quality of the refinements is good, since $R_{wp}\geq \;R_{exp}$, $\chi^{2}={\left(\frac{R_{wp}}{R_{exp}}\right)}^{2}$ is higher than one, and the goodness of fit (GoF) given by $GoF=\sqrt{\chi^{2}}$ is close to one [37].

### 3.3. Morphological Characterization

**Figure 5:**
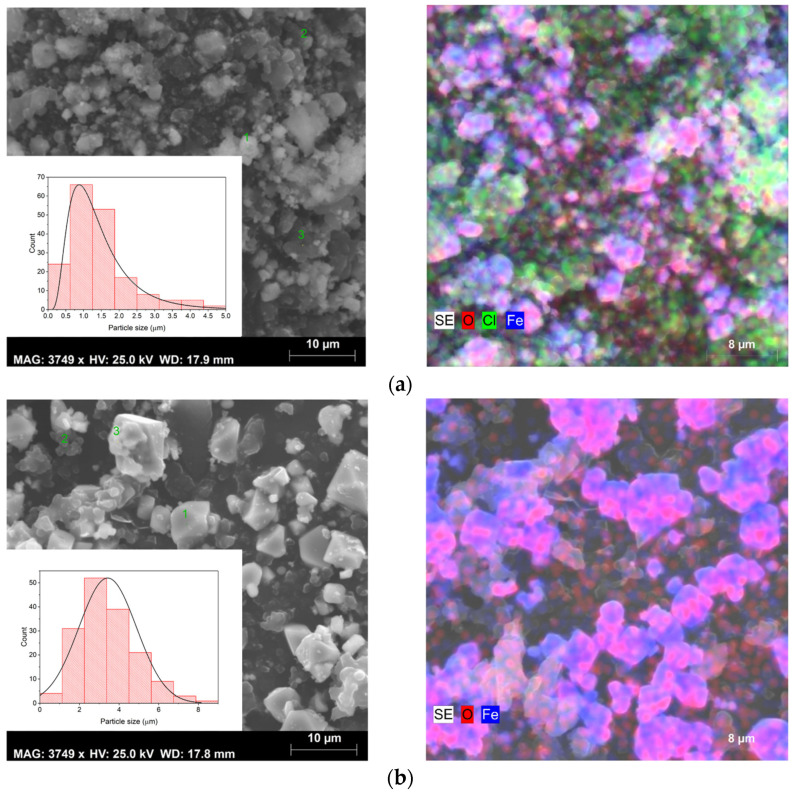

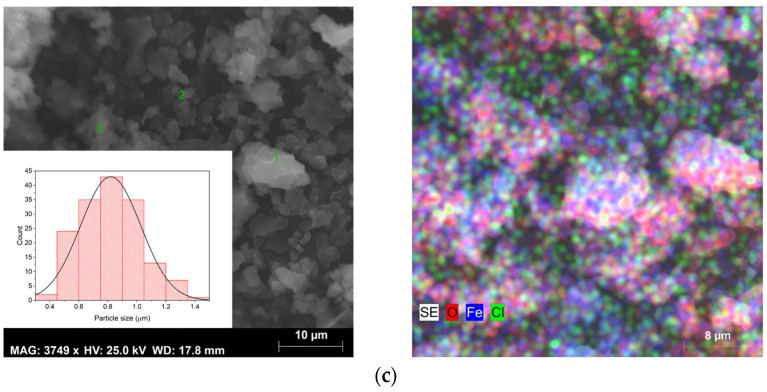
SEM images, size distribution (**left** figures), and elementary mapping obtained by EDS (**right** figures) of sample (**a**) S1, (**b**) S2, and (**c**) S3.

Moving on to the morphological characterization (Figure 5), an examination of the SEM images reveals that with the increase in temperature, the grain size also increases. The average grain size was determined using ImageJ software, based on 160 measurements per sample; subsequently, a histogram was plotted according to the Sturges method [38]. The mean grain size was determined using a normal distribution function for samples S2 and S3 while a log-normal distribution function was employed for fitting sample S1. The calculated mean grain size was 1.2 ± 0.9 µm for sample S1, 3.4 ± 1.5 µm for sample S2, and 0.8 ± 0.2 µm for sample S3, respectively. In samples S1 and S3, the presence of grain coalescence phenomena resulted in a less accurate determination of the average grain size. All samples exhibited a micrometer range, suggesting that the particles synthesized require reduction to an ideal size between 10 and 100 nm for potential applications in magnetic hyperthermia [39,40]. One viable way to achieve this size range is through ball milling, as performed by B. Costa et al. [30]. However, the process of particle size reduction via this technique must be carefully managed to address potential limitations, such as the formation of an amorphous phase. Thus, a detailed examination of the impact of milling parameters, with a particular focus on milling time and speed, should be conducted to guarantee an effective reduction in particle size while simultaneously reducing the risk of undesirable structural changes [30].

For sample S1, SEM images reveal the existence of different grain habits, due to the presence of several crystalline phases, as can be seen in the results obtained by XRD (Figure 3). However, the existence of varied phases combined with the phenomenon of coalescence makes it difficult to identify the phase corresponding to each type of grain. In sample S2, it is possible to observe only octahedral grains with different sizes, which is characteristic of magnetite [41]. Sample S3 presents an irregular shape due to the agglomeration of the grains [42].

SEM combined with EDS allowed for an elementary analysis of the samples. The results acquired are shown in Figure 5 (right micrographs), where the carbon element resulting from the deposition before this analysis has been excluded. EDS confirmed the presence of the main elements oxygen (O) and iron (Fe) in all samples. However, in samples S1 and S3, the element chlorine (Cl) was also detected, which may be linked to the iron chlorides used as raw materials in the sol–gel synthesis method. As evidenced in the XRD results (Figure 3) in sample S1, the chlorides were detected as a crystalline phase and also identified by SEM-EDS, while in sample S3 they probably are in an amorphous phase, being only detected in the elementary analysis (Figure 5), corroborating the results of both techniques.

All samples exhibited heterogeneous composition, except sample S3. Therefore, elementary analysis was performed at different points (grains) in each sample. The results are presented in Table 3.

### 3.4. Magnetic Characterization

#### 3.4.1. Magnetic Susceptibility

**Figure 6:**
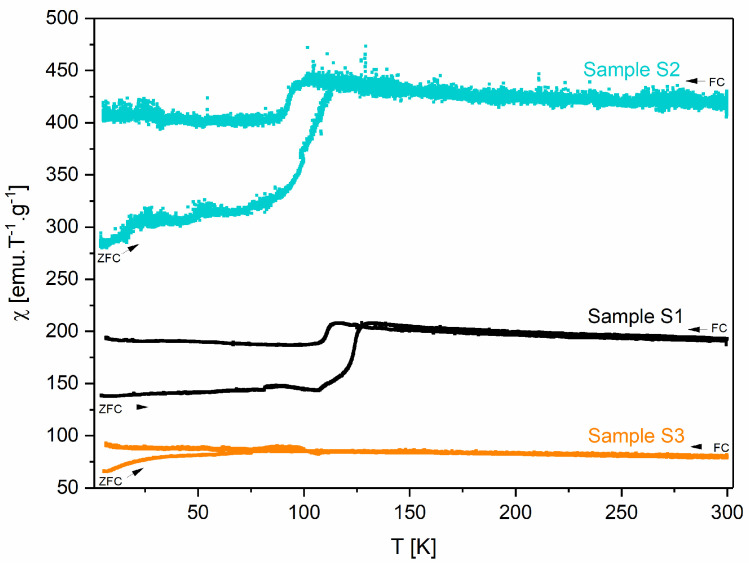
Magnetic susceptibility as a function of the temperature of samples S1, S2, and S3.

Regarding the magnetic characterization, Figure 6 shows the graphs of magnetic susceptibility as a function of the temperature of samples S1, S2, and S3. All the samples exhibit a magnetic transition between 100 and 115 K, which can be attributed to the Verwey transition [43,44,45]. Among all the samples, at room temperature, S2 is the one that exhibits the highest magnetic susceptibility (χ = 423 emu.T^−1^.g^−1^; H = 1 kOe) due to the pure magnetite phase in its composition being in accordance with the literature [46].

The blocking temperature (T_B_) for each sample is presented in Table 4. This value corresponds to the maximum of the ZFC (zero-field-cooled) curve [47]. However, it should be noted that for samples S1 and S2, this value could be masked by the Verwey transition, with its determination being more difficult.

#### 3.4.2. Hysteresis Loop

**Figure 7:**
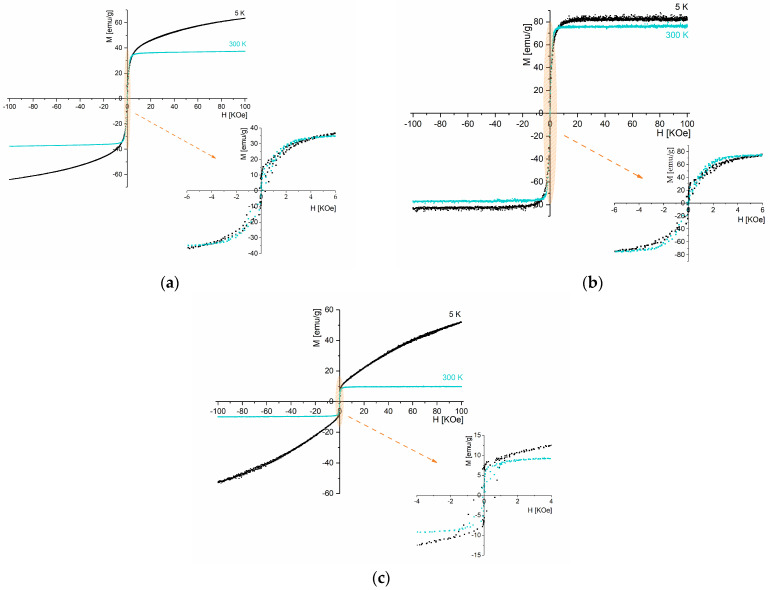
Hysteresis loop at 5 K and 300 K for samples (**a**) S1, (**b**) S2, and (**c**) S3.

Figure 7 shows the hysteresis curves for samples S1, S2, and S3 at 5 and 300 K and Table 5 presents the magnetization, coercive field, and remanent magnetization obtained by analyzing these curves.

Among the samples, S2 exhibits the highest magnetization, with a saturation magnetization of 76 emu/g under a 7 kOe magnetic field at 300 K. Considering that sample S2 contains 100% of monoclinic Fe_3_O_4_ crystalline phase, the saturation magnetization value obtained is slightly lower than the literature-reported value (Ms bulk = 92 emu/g) [48].

At room temperature, sample S1 displays a lower saturation magnetization (37 emu/g) than S2. According to the XRD (Figure 3, Table 2), this sample presents other phases besides the crystalline Fe_3_O_4_ phase, including FeCl_2_·4H_2_O, FeO(OH), and α-Fe_2_O_3_. The saturation magnetization reported in the literature at 300 K for α-Fe_2_O_3_ is 0.3 emu/g [49] and for FeO(OH) is 3.46 emu/g [50]. Hence, the decrease in magnetization can be attributed to the presence of other phases than magnetite.

Sample S3 also presents only Fe_3_O_4_; however, its saturation magnetization (10 emu/g, 300 K) is much lower than sample S2 (76 emu/g, 300 k). The enhanced magnetic properties of sample S2 can be related to its higher degree of crystallinity found on X-ray diffraction analysis (Figure 3) [51].

At room temperature, although reduced, magnetic hysteresis is visible in all samples, suggesting a dominant ferrimagnetic behavior [52]. This behavior is also observed in sample S2 at 5 K.

#### 3.4.3. Specific Absorption Rate

Magnetic hyperthermia measurements were performed to evaluate the heating capacity of the three samples. The results obtained are shown in Table 6.

The SAR values of these samples are directly related to their ferrimagnetic behavior observed in the hysteresis loops (Figure 7). The micrometer size of the particles contributes to the reduced SAR values presented in Table 6, since smaller nanoparticles typically exhibit higher surface area and enhanced heat dissipation.

According to the data in Table 6, samples S1 and S2 demonstrated the highest thermal efficiency, while sample S3 showed the worst SAR value. Although sample S3 consists entirely of magnetite, it also contains an amorphous phase (Figure 3) and displays signs of grain agglomeration (Figure 5), both of which negatively impact the SAR value [53,54].

As evidenced in SEM results (Figure 5), the nanoparticles produced using this method need size reduction to be applied in magnetic hyperthermia. Reducing the particle size to the nanometer scale will impact the SAR value, as smaller particles typically exhibit higher SAR values, enhancing their heating efficiency [55]. A higher SAR value enables a greater heating capacity of the nanoparticles, reducing the concentration needed for therapeutic applications, and thereby minimizing potential side effects in patients [4].

### 3.5. Cytotoxicity Analysis

**Figure 8:**
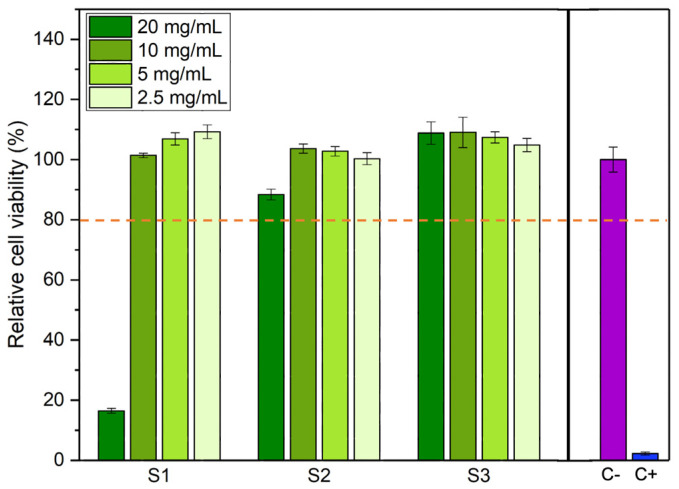
Relative cell viability for different concentrations of each sample. Cell viability above 80% indicates that the sample is not cytotoxic (orange dashed line). C-: negative control. C+: positive control.

The results of the cell viability tests are shown in Figure 8. Samples that lead to a relative cell viability above 80% are considered non-cytotoxic and, therefore, can be considered safe for use in the human body [32]. Thus, the cytotoxicity analysis reveals that at concentrations below 10 mg/mL, all samples present a non-cytotoxic behavior. At a concentration of 20 mg/mL, only sample S1 displays cytotoxic behavior. Since cells are never in contact with the powdered samples, cytotoxicity must arise from either the dissolution of ions from the iron oxide particles or the capture of ions or other medium constituents by the iron oxide particles that causes an imbalance in medium osmolality or a depletion of necessary nutrients that lead to cell death.

In addition to cytotoxicity assays, it would be interesting in future work to study oxidative stress, since these particles, due to their iron content, are capable of participating in the Fenton reaction, which generates reactive oxygen species (ROS) that induce oxidative damage in tumor cells. The combination of these mechanisms with their heat capacity produces a synergistic effect in cancer treatment [56].

## 4. Conclusions

This study aimed to synthesize magnetite nanoparticles for potential applications in magnetic hyperthermia through a sustainable coconut water-assisted sol–gel method. The pure phase of magnetite was successfully obtained in the samples treated at 700 and 400 °C (washed powder) for 4 h under an atmosphere of argon. The washing process before the heat treatment removes chlorides and nutrients from the coconut water precursor, facilitating the formation of magnetite at a lower temperature. This process reduces energy consumption, making it a more efficient method for obtaining magnetite. All synthesized samples exhibited micrometer-scale sizes, resulting in ferrimagnetic behavior characteristics of magnetite above the critical size. The saturation magnetization values were 37.33 (350 °C, 48 h), 75.88 (700 °C, 4 h), and 9.70 (washed powder, 400 °C, for 4 h) emu/g at 300 K and the respective SAR values were 27.1, 19.9, and 14.1 W/g. Regarding cytotoxicity, the samples appear non-toxic at concentrations below 10 mg/mL.

Despite the interesting results, the high particle size obtained through this approach makes these particles unsuitable for magnetic hyperthermia applications. Thus, in the future, the particle size must be reduced, for example, through ball milling, to minimize the risk of obstructing blood vessels. Furthermore, more in vitro tests are essential to evaluate their therapeutic potential. These tests should include stability studies in physiological environments, assessments of ROS generation, and experiments on cancer cell lines to determine their cytotoxicity and efficacy. Following these preliminary evaluations, in vivo studies using animal models will be necessary to further validate their safety and effectiveness.

## Figures and Tables

**Table 1:** Sample names.

Sample	Heat Treatment Temperature of Base Powder and Conditions
S0	700 °C, for 4 h; heating rate of 5 °C/min; in normal atmosphere
S1	350 °C, for 48 h; heating rate of 5 °C/min; in argon atmosphere
S2	700 °C, for 4 h; heating rate of 5 °C/min; in argon atmosphere
S3	400 °C, for 4 h; heating rate of 5 °C/min; in argon atmosphere (washed powder)

**Table 2:** Crystalline phases, crystalline system, and weight percentage (wt%) for the heat-treated samples. * Due to the high number of phases present in the sample, it was not possible to obtain the percentage of each phase in this sample.

Sample	Crystalline Phase	Crystalline System	wt%
S0	Fe_2_O_3_	Rhombohedral	100%
S1	Fe_3_O_4_	Cubic	*
	α-Fe_2_O_3_	Rhombohedral	
	FeO(OH)	Orthorhombic	
	FeCl_2_·4H_2_O	Monoclinic	
	FeCl_2_·4H_2_O	Unknown	
S2	Fe_3_O_4_	Monoclinic	100%
S3	Fe_3_O_4_	Cubic	100%

**Table 3:** Elementary analysis of samples S1, S2, and S3 obtained by EDS.

Sample		S1	S2	S3
Grain	Element	Atomic Percentage (%_at_)		
1	O	74.01	68.68	89.53
	Fe	25.70	31.32	9.82
	Cl	0.30	-	0.65
2	O	45.80	70.30	66.52
	Fe	50.88	29.79	30.42
	Cl	3.33	-	3.05
3	O	67.85	66.59	85.83
	Fe	24.52	33.41	13.08
	Cl	7.63	-	1.09

**Table 4:** Blocking temperatures of samples S1, S2, and S3.

Sample	S1	S2	S3
T_B_ (K)	133	117	87

**Table 5:** Saturation magnetization, coercive field, and remanent magnetization for samples S1, S2, and S3 at 5 and 300 k.

T (K)	Sample	Magnetization at 100 kOe (emu/g)	Coercive Field (kOe)	Remanent Magnetization (emu/g)
5	S1	63.45	0.266	5.60
	S2	83.54	0.032	4.93
	S3	51.91	0.544	14.51
300	S1	37.33	0.051	6.07
	S2	75.88	0.015	0.75
	S3	9.70	0.016	2.23

**Table 6:** Results obtained for temperature variation after 650 s and specific absorption rate of samples S1, S2, and S3.

Sample	∆T (°C); ∆t = 650 s	SAR (W/g)
S1	20.3 ± 0.7	27.1 ± 5.0
S2	14.5 ± 2.1	19.9 ± 4.2
S3	9.57 ± 1.19	14.1 ± 3.3

## Data Availability

The original contributions presented in the study are included in the article, further inquiries can be directed to the corresponding author.
